# Supplementary material for: Myosin-based regulation of twitch and tetanic contractions in mammalian skeletal muscle
Source: eLife. 2021 Jun 14;10:e68211. doi: 10.7554/eLife.68211 (PMC8275128; doi:10.7554/eLife.68211)
Supplement: Transparent reporting form [file elife-68211-transrepform.docx]

***eLife’s* transparent reporting form**

We encourage authors to provide detailed information *within their submission* to facilitate the interpretation and replication of experiments. Authors can upload supporting documentation to indicate the use of appropriate reporting guidelines for health-related research (see [EQUATOR Network](http://www.equator-network.org/%20)), life science research (see the [BioSharing Information Resource](https://biosharing.org/)), or the [ARRIVE guidelines](http://www.plosbiology.org/article/info:doi/10.1371/journal.pbio.1000412) for reporting work involving animal research. Where applicable, authors should refer to any relevant reporting standards documents in this form.

If you have any questions, please consult our Journal Policies and/or contact us: [editorial@elifesciences.org](mailto:editorial@elifesciences.org).

**Sample-size estimation**

- You should state whether an appropriate sample size was computed when the study was being designed
- You should state the statistical method of sample size computation and any required assumptions
- If no explicit power analysis was used, you should describe how you decided what sample (replicate) size (number) to use

Please outline where this information can be found within the submission (e.g., sections or figure legends), or explain why this information doesn’t apply to your submission:

No power analyses were conducted to determine sample sizes given that time-resolved X-ray diffraction studies of skeletal muscle are constrained by the limited availability of synchrotron beamlines. We aimed for a minimum of four experiments for both twitch and tetanus protocols, where repeated X-ray exposures were performed in each muscle and averaged for each muscle, thus increasing the number of replicates. Replicates are discussed in the next section. Such an approach is commonplace for X-ray diffraction studies, where n = 4 muscles have previously been published (e.g. Brunello *et al*., 2006, 2009). To reduce the use of animals (NC3R’s), we performed multiple protocols in each preparation to the maximum extent allowed by radiation damage. Information regarding n-values can be found in the sections titled *Muscle Preparation* and *X-ray Data Collection*.

**Replicates**

- You should report how often each experiment was performed
- You should include a definition of biological versus technical replication
- The data obtained should be provided and sufficient information should be provided to indicate the number of independent biological and/or technical replicates
- If you encountered any outliers, you should describe how these were handled
- Criteria for exclusion/inclusion of data should be clearly stated
- High-throughput sequence data should be uploaded before submission, with a private link for reviewers provided (these are available from both GEO and ArrayExpress)

Please outline where this information can be found within the submission (e.g., sections or figure legends), or explain why this information doesn’t apply to your submission:

The twitch protocol was performed four times in four separate muscles, whilst the tetanus protocol was performed five times in five separate muscles. In two muscles, both the twitch and tetanus protocols were performed, meaning a total of seven animals were used. The inbred C57BL/6J mouse was chosen to ensure homogenous biological replication of our results.

Technically, X-ray exposures were provided to contracting muscle as many times as possible before the quality of the preparation declined substantially. Each 2D X-ray pattern was manually examined and those of poor quality (e.g. presence of strongly diffracting collagen regions, “splitting” of the equatorial-based reflections) were not included in the analyses. Therefore, data for the twitch protocol was derived from 8-26 replicates per muscle and from 6-12 replicates in the tetanus protocol.

To determine quality of the data derived from integrations of the 2D X-ray patterns and identify any outliers, individual data points were plotted from each experiment in each protocol.

**Statistical reporting**

- Statistical analysis methods should be described and justified
- Raw data should be presented in figures whenever informative to do so (typically when N per group is less than 10)
- For each experiment, you should identify the statistical tests used, exact values of N, definitions of center, methods of multiple test correction, and dispersion and precision measures (e.g., mean, median, SD, SEM, confidence intervals; and, for the major substantive results, a measure of effect size (e.g., Pearson's r, Cohen's d)
- Report exact p-values wherever possible alongside the summary statistics and 95% confidence intervals. These should be reported for all key questions and not only when the p-value is less than 0.05.

Please outline where this information can be found within the submission (e.g., sections or figure legends), or explain why this information doesn’t apply to your submission:

All data are presented as mean ± standard error.

To determine whether there were significant differences in the half-time for changes in X-ray structures in relation to force in a given protocol, a two-tailed t-test was performed with significance set at P<0.05. t-tests were performed for the rise of force in the tetanus, and during relaxation in the twitch and tetanus protocols. All t-tests were performed on n = 5 for the tetanus and n = 4 for the twitch. Data pertaining to these results are provided in Table 1 of the manuscript. This statistical test was chosen as ANOVA’s were not appropriate for our analyses as we were interested in when significant differences between an X-ray parameter and force occurred within the *same* protocol, and not between the two protocols or between different X-ray parameters.

We have opted not to include the individual P-values as, in many cases, P<0.001. Instead, we have used asterisk symbols to denote the level of significance in Table 1, where * = P<0.05; ** = P<0.001; *** = P<0.0001. Exact P-values can be provided if necessary.

(For large datasets, or papers with a very large number of statistical tests, you may upload a single table file with tests, Ns, etc., with reference to sections in the manuscript.)

**Group allocation**

- Indicate how samples were allocated into experimental groups (in the case of clinical studies, please specify allocation to treatment method); if randomization was used, please also state if restricted randomization was applied
- Indicate if masking was used during group allocation, data collection and/or data analysis

Please outline where this information can be found within the submission (e.g., sections or figure legends), or explain why this information doesn’t apply to your submission:

Animals were selected at random from cages containing 4-5 animals per cage. Each protocol was performed in a randomized order.

**Additional data files (“source data”)**

- We encourage you to upload relevant additional data files, such as numerical data that are represented as a graph in a figure, or as a summary table
- Where provided, these should be in the most useful format, and they can be uploaded as “Source data” files linked to a main figure or table
- Include model definition files including the full list of parameters used
- Include code used for data analysis (e.g., R, MatLab)
- Avoid stating that data files are “available upon request”

Please indicate the figures or tables for which source data files have been provided:

Source data are provided for figures 1 to 6.
